# Global burden of head and neck cancer from 1990 to 2021: A comprehensive analysis and projections to 2030 based on the global burden of disease study 2021

**DOI:** 10.1371/journal.pone.0330805

**Published:** 2025-09-08

**Authors:** Muling Deng, Yuhao Lin, Linghui Yan, Zhaodong Fei, Chuanben Chen, Jianming Ding

**Affiliations:** Department of Radiation Oncology, Clinical Oncology School of Fujian Medical University, Fujian Cancer Hospital, Fuzhou, Fujian, China; The Chinese University of Hong Kong, HONG KONG

## Abstract

**Background:**

Head and neck cancer (HNC) is a significant global health concern with rising incidence and mortality in certain regions. This study aimed to evaluate the global burden and temporal trends of HNC from 1990 to 2021 and to project its future burden through 2030.

**Methods:**

Data were obtained from the Global Burden of Disease (GBD) 2021 study. Joinpoint regression was used to assess temporal trends in age-standardized incidence rates (ASIR), age-standardized death rates (ASDR), and disability-adjusted life years (DALYs). Age–period–cohort (APC) and Bayesian APC (BAPC) models were applied to evaluate age, period, and cohort effects and to project future trends. Decomposition analysis was conducted to explore the contributions of population aging, growth, and epidemiological changes.

**Results:**

In 2021, there were 792,280 new HNC cases and 424,066 deaths globally. Age-standardized incidence rates remained stable, while death rates and DALYs significantly declined. Incidence rose notably in East Asia, whereas mortality and DALYs increased substantially in Oceania. Gender differences were evident, with higher burdens in males, although female incidence rates recently increased. Aging and population growth were key contributors to the rising burden. Projections suggest a notable increase in female incidence and continued decline in male DALYs by 2030. Among HNC subtypes, lip and oral cavity cancers had the highest burden, whereas other pharyngeal cancers showed increasing incidence trends.

**Conclusions:**

Despite overall declines in mortality and disease burden, the global incidence of HNC remains substantial. Targeted interventions—such as tobacco and alcohol control and widespread HPV vaccination—are essential to mitigate the future burden of HNC.

## Introduction

Head and neck cancer (HNC) is a prevalent malignant tumor, primarily encompassing cancers of the lip, oral cavity, and larynx. It originates from diverse anatomical sites within the head and neck region, particularly the epithelial linings of the oral cavity, pharynx, and larynx [1,2]. HNC ranks as the seventh most prevalent cancer worldwide and is frequently characterized by high aggressiveness, metastatic potential, recurrence, and therapeutic resistance [3–5]. Its incidence varies by anatomical tumor site and geographical distribution [6]. The predominant malignant subtype of HNC is head and neck squamous cell carcinoma (HNSCC) [7]. In 2022, HNC was diagnosed in 946,456 new cases, resulting in 482,001 deaths, with HNSCC comprising over 90% of all cases [8,9]. The incidence of HNC exhibits substantial geographical variability, with particularly high rates observed in Southeast Asia [6]. A marked gender disparity is evident in HNC, with incidence rates in men being two to four times higher than in women [10]. However, recent studies indicate that the incidence rate among women has been rising at twice the rate observed in men [11].The primary risk factors for HNC encompass tobacco use, excessive alcohol consumption, human papillomavirus (HPV) infection, betel nut chewing, oral sex, mechanical irritation, and occupational exposures [8,12]. In recent years, the incidence of HNC attributable to detrimental behaviors such as smoking and excessive alcohol consumption has declined, whereas HPV-related HNC cases, particularly those associated with HPV-16, have been increasing [13,14]. Patients with HNC frequently suffer from dysphagia, respiratory distress, and cervical fibrosis, along with psychological disorders such as anxiety and depression, thereby imposing substantial burdens on healthcare systems [15–17]. The management of HNC typically involves a multidisciplinary approach comprising surgery, radiotherapy, chemotherapy, and targeted therapies [9].

Previous small-sample studies on the disease burden of HNC have been limited by sociological factors [18], including demographic variations—such as age distribution, racial composition, gender disparities, and economic conditions—across different regions [3]. These limitations impede a comprehensive understanding of the global disease burden of HNC. The Global Burden of Disease (GBD) database utilizes standardized methodologies to ensure consistency and comparability in assessing disease burden across different populations and time periods, offering valuable resources for global disease burden evaluation. In this study, we employed Joinpoint Regression analysis to identify significant trends in the incidence, mortality, and disability-adjusted life years (DALYs) associated with HNC. Additionally, we conducted Age-Period-Cohort (APC) analysis to assess the effects of age, time period, and birth cohort on the HNC burden. Furthermore, we utilized Bayesian Age-Period-Cohort (BAPC) analysis to project future trends in the HNC burden through 2030 and performed a decomposition analysis to evaluate the relative contributions of aging, epidemiological changes, and population growth to the HNC burden.

## Methods

### 2.1 Data source

This study is part of the Global Burden of Disease (GBD) 2021 project, which evaluated 369 diseases and injuries [19]. The GBD 2021 study analyzed a total of 88 risk factors organized hierarchically, including an aggregated all-risk measure, 3 level-1 risks (environmental and occupational, behavioral, and metabolic risks), 20 level-2 risks, 42 level-3 risks, and 22 level-4 risks.The complete GBD 2021 risk factor hierarchy is provided in S2 Table. Data on head and neck cancer (HNC) from 1990 to 2021 were obtained from the Global Health Data Exchange (GHDx) query tool (https://ghdx.healthdata.org/gbd-results-tool). We extracted estimates of incidence, mortality, and disability-adjusted life years (DALYs), disaggregated by sex (both sexes, male, and female) and age (19 age groups from <5 to ≥95 years, in 5-year intervals).

All estimates in the GBD 2021 study are generated using standardized statistical modeling frameworks, and are reported with 95% uncertainty intervals (UIs), based on the 2.5th and 97.5th percentiles from 1,000 posterior draws. This study adheres to the Guidelines for Accurate and Transparent Health Estimates Reporting (GATHER) to ensure methodological rigor and transparency in health data analysis. [20].

### 2.2 Study design

We conducted a cross-sectional analysis to evaluate the burden, risk factors, components, and health disparities related to HNC. The outcomes measured were number, deaths, incidence, age-standardized rates of incidence (ASIR), age-standardized death rates (ASDR), disability-adjusted life years (DALYs), and age-standardized DALYs.

### 2.3 Statistical analysis

The estimated average percentage change (EAPC) was computed to assess the trends in the age-standardized rates (ASRs) of HNC burdens. A positive EAPC and its 95% confidence interval (CI) lower bound >0 indicated an increasing trend; both values <0 indicated a decreasing trend; values including 0 suggested a stable trend. To identify significant changes in HNC trends over time, joinpoint regression analysis was performed. This method fits a sequence of connected straight lines to the trend data, enabling the detection of slope changes at specific time points. The annual percentage change was then calculated to quantify these trends, which helped pinpoint periods of increase or decrease in HNC metrics [21].

APC analysis was employed to quantify the separate effects of age, time period, and birth cohort on HNC incidence, and DALY rates. To address the collinearity inherent in APC models, we applied the intrinsic estimator (IE) method, which offers more stable and interpretable coefficient estimates. Decomposition analysis breaks down changes in HNC metrics, revealing the forces behind trends in incidence, deaths, and DALYs. This approach quantified the contributions of aging, population growth, and epidemiological changes to HNC burden shifts.

BAPC analysis, combined with integrated nested Laplace approximation, was used to project future trends in HNC incidence, deaths, and DALYs up to 2030, forecasting trends based on historical data. Data analyses were conducted using R (version 4.2.2) and R Studio software. A two-sided P-value <0.05 was considered statistically significant.

## Results

The global burden of HNC from 1990 to 2021, including incidence, deaths, DALYs, ASIR, ASDR, age-standardized DALYs, and EAPC, was summarized in Table 1. In 2021, the global incidence of HNC was 792,280 (95% uncertainty interval (UI): (736,667−845,584)), with an ASIR 9.1 (95% UI: 8.47–9.72). The number of deaths was 424,066 (95% UI: 392,210−455,599), resulting in an ASDR of 4.9 (95% UI: 4.54–5.26). The number of DALYs was 11,861,160 (95% UI: 10,872,037−12,794,114), and age-standardized DALYs was 135.89 (95% UI: 124.46–146.58). Between 1990 and 2021, the global ASIR for HNC remained stable (EAPC: 0.01; 95% CI: −0.06 to 0.08). In contrast, significant declines were observed in both ASDR (EAPC: −0.58; 95% CI: −0.65 to −0.51) and age-standardized DALYs (EAPC: −0.67; 95% CI: −0.74 to −0.60). Among the 21 GBD regions, East Asia demonstrated the greatest increase in ASIR (EAPC: 0.92; 95% CI: 0.75–1.09), while Oceania reported the highest increases in ASDR (EAPC: 0.31; 95% CI: 0.25–0.37) and age-standardized DALYs (EAPC: 0.34; 95% CI: 0.27–0.40). Central Asia and Central Latin America exhibited the largest declines in ASIR, with EAPCs of −1.31 (95% CI: −1.44 to −1.18) and −1.31 (95% CI: −1.42 to −1.19), respectively. Southern Latin America experienced the most substantial reductions in ASDR (EAPC: −1.95; 95% CI: −2.04 to −1.85) and DALYs (EAPC: −2.26; 95% CI: −2.35 to −2.16).

**Table 1:** Global Burden of Head and Neck Cancer and Trends from 1990 to 2021 by 21 GBD regions,5 SDI regions, 4 World bank regions and Gender.

	1990		2021			1990		2021			1990		2021		
	Incidence cases	ASIR	Incidence cases	ASIR	EAPC	Death cases	ASDR	Death cases	ASDR	EAPC	DALYs cases	Age_standardised DALYs	DALYs cases	Age_standardised DALYs	EAPC
Characteristics	(95%UI)	per100,000 (95%UI)	(95%UI)	per100,000 (95%UI)	(95%UI)	(95%UI)	per100,000 (95%UI)	(95%UI)	per100,000 (95%UI)	(95%UI)	(95%UI)	per100,000 (95%UI)	(95%UI)	per100,000 (95%UI)	(95%UI)
**Global**	363781 (347214,382181)	8.89 (8.48,9.34)	792280 (736667,845584)	9.1 (8.47,9.72)	0.01 (−0.06 to 0.08)	227536 (213909,241759)	5.68 (5.34,6.04)	424066 (392210,455599)	4.9 (4.54,5.26)	−0.58 (−0.65 to −0.51)	6784525 (6378226,7223444)	160.87 (151.31,171.23)	11861160 (10872037,12794114)	135.89 (124.46,146.58)	−0.67 (−0.74 to −0.6)
**Gender**															
**Female**	82999 (75399, 89519)	3.86 (3.5,4.15)	210507 (189546,233681)	4.63 (4.17,5.14)	0.52 (0.45 to 0.6)	49113 (43249,54137)	2.32 (2.06,2.55)	106346 (94360,120750)	2.32 (2.07,2.63)	−0.1 (−0.17 to −0.03)	1408837 (1227262,1565479)	64.14 (55.96,71.24)	2863272 (2548814,3263622)	63.89 (56.76,72.9)	−0.13 (−0.22 to −0.05)
**Male**	280783 (266351,297169)	14.61 (13.85,15.46)	581772 (532939,628516)	14.07 (12.9,15.19)	−0.19 (−0.26 to −0.11)	178423 (166617,191924)	9.62 (8.98,10.34)	317720 (287966,345333)	7.82 (7.1,8.48)	−0.77 (−0.84 to −0.7)	5375689 (5014226,5793681)	266.3 (248.56,286.46)	8997888 (8066769,9814328)	213.45 (191.58,232.58)	−0.83 (−0.91 to −0.76)
**SDI_regions**															
**Low SDI**	18126 (14970,21535)	7.49 (6.22,8.87)	43935 (37238,51290)	8.09 (6.92,9.37)	0.13 (0.03 to 0.23)	15315 (12638,18250)	6.63 (5.48,7.88)	33723 (28583,39281)	6.59 (5.64,7.64)	−0.1 (−0.17 to −0.03)	477174 (392761,569093)	183.73 (151.55,219.48)	1031983 (867044,1209958)	175.98 (148.91,205.49)	−0.26 (−0.33 to −0.19)
**Low-middle SDI**	65720 (57437,74903)	10.06 (8.79,11.44)	178213 (157588,198486)	11.77 (10.44,13.09)	0.48 (0.4 to 0.56)	54328 (47192,62183)	8.66 (7.52,9.9)	130571 (115829,145497)	8.95 (7.96,9.96)	0.08 (0.03 to 0.13)	1694374 (1474864,1941206)	243.09 (211.13,278.34)	3899011 (3411631,4366834)	246.93 (217.47,275.63)	0.04 (−0.01 to 0.08)
**Middle SDI**	69019 (63973,73869)	6.37 (5.92,6.82)	206185 (185496,227961)	7.47 (6.72,8.25)	0.43 (0.33 to 0.53)	52837 (48794,56697)	5.14 (4.76,5.5)	121753 (109626,134110)	4.52 (4.07,4.98)	−0.53 (−0.59 to −0.47)	1597779 (1476813,1718188)	138.45 (128.07,148.77)	3413507 (3062835,3763074)	121.03 (108.78,133.31)	−0.56 (−0.62 to −0.5)
**High-middle SDI**	90196 (86332,94191)	8.81 (8.42,9.21)	156155 (142289,169979)	7.93 (7.23,8.63)	−0.49 (−0.6 to −0.39)	56255 (53582,59087)	5.6 (5.33,5.88)	71599 (65474,77873)	3.61 (3.3,3.93)	−1.65 (−1.74 to −1.56)	1665525 (1589345,1748641)	160.52 (153.2,168.54)	1922689 (1758654,2092998)	98.31 (89.98,106.9)	−1.86 (−1.97 to −1.76)
**High SDI**	120271 (116133,123681)	11.26 (10.9,11.59)	207020 (194121,216506)	10.76 (10.19,11.23)	−0.14 (−0.19 to −0.08)	48496 (46632,49975)	4.5 (4.32,4.63)	65993 (60965,69507)	3.2 (3,3.37)	−1.13 (−1.21 to −1.05)	1340800 (1295973,1383957)	127.92 (123.68,132.04)	1582553 (1493129,1661582)	85.11 (80.65,89.2)	−1.36 (−1.42 to −1.31)
**GBD regions**															
**Andean Latin America**	626 (546,714)	3.02 (2.63,3.45)	1615 (1276,2016)	2.71 (2.15,3.38)	−0.39 (−0.63 to −0.15)	524 (457,598)	2.62 (2.29,3.01)	1046 (825,1292)	1.79 (1.41,2.22)	−1.29 (−1.5 to −1.08)	14256 (12407,16250)	64.89 (56.41,74.1)	26231 (20617,32535)	43.31 (34.04,53.68)	−1.4 (−1.62 to −1.18)
**Australasia**	2810 (2577,3056)	12.21 (11.19,13.26)	5766 (5077,6459)	11.71 (10.33,13.09)	−0.14 (−0.35 to 0.07)	891 (823,965)	3.83 (3.54,4.15)	1342 (1182,1502)	2.51 (2.21,2.79)	−1.4 (−1.66 to −1.15)	23586 (21734,25676)	103.19 (94.9,112.25)	31827 (28231,35582)	65.11 (58.03,72.71)	−1.5 (−1.74 to −1.25)
**Caribbean**	2483 (2307,2679)	9.56 (8.88,10.3)	5138 (4425,5932)	9.5 (8.18,10.94)	0.18 (0.08 to 0.28)	1776 (1647,1923)	6.98 (6.47,7.54)	3239 (2803,3751)	5.98 (5.17,6.92)	−0.3 (−0.41 to −0.19)	46075 (42598,50265)	174.17 (161.15,189.95)	82749 (70648,97109)	153.07 (130.73,179.61)	−0.21 (−0.32 to −0.09)
**Central Asia**	3447 (3246,3686)	6.97 (6.54,7.46)	4093 (3599,4652)	4.7 (4.14,5.32)	−1.31 (−1.44 to −1.18)	2684 (2525,2872)	5.52 (5.18,5.93)	2800 (2464,3174)	3.34 (2.94,3.77)	−1.7 (−1.82 to −1.59)	82706 (78066,88159)	162.17 (152.95,172.97)	83319 (72816,95115)	91.57 (80.26,104.19)	−1.98 (−2.1 to −1.86)
**Central Europe**	16894 (16083,17812)	11.29 (10.75,11.89)	28146 (25733,30609)	14.04 (12.83,15.31)	0.63 (0.53 to 0.73)	11954 (11386,12579)	8.02 (7.63,8.42)	15577 (14240,16894)	7.51 (6.87,8.16)	−0.3 (−0.37 to −0.23)	361019 (344074,380421)	242.12 (230.85,255.07)	419695 (382429,457151)	216.7 (197.34,236.17)	−0.5 (−0.59 to −0.4)
**Central Latin America**	3797 (3661,3921)	4.62 (4.44,4.77)	8542 (7557,9644)	3.4 (3.01,3.85)	−1.31 (−1.42 to −1.19)	3050 (2938,3151)	3.89 (3.73,4.02)	5638 (4989,6365)	2.29 (2.03,2.58)	−2 (−2.11 to −1.9)	80007 (77350,82502)	91.41 (88.19,94.37)	139654 (123370,158680)	54.74 (48.37,62.15)	−1.98 (−2.09 to −1.87)
**Central Sub-Saharan Africa**	934 (694,1221)	4 (3.08,5.11)	2364 (1775,3048)	4.02 (3.03,5.16)	0 (−0.17 to 0.17)	808 (603,1059)	3.67 (2.85,4.66)	1890 (1411,2448)	3.45 (2.59,4.47)	−0.2 (−0.33 to −0.07)	24757 (18246,32564)	96.65 (72.62,125.65)	58977 (43755,77118)	90.5 (67.62,117.07)	−0.21 (−0.33 to −0.08)
**East Asia**	37467 (31490,43604)	4.19 (3.55,4.86)	117844 (95107,145259)	5.34 (4.32,6.54)	0.92 (0.75 to 1.09)	28256 (23661,32943)	3.34 (2.83,3.87)	53730 (43261,66170)	2.47 (2.01,3.03)	−0.99 (−1.11 to −0.87)	825303 (686820,966793)	87.14 (72.9,101.81)	1391856 (1111302,1723171)	62.8 (50.34,77.3)	−1.09 (−1.21 to −0.96)
**Eastern Europe**	32251 (30913,34068)	11.42 (10.94,12.07)	43175 (38747,47778)	13.09 (11.77,14.49)	0.03 (−0.21 to 0.27)	20419 (19591,21483)	7.22 (6.93,7.6)	20713 (18576,23087)	6.07 (5.45,6.77)	−1.09 (−1.29 to −0.89)	635775 (610124,672002)	225.94 (216.73,239.13)	605915 (541238,676751)	185.76 (165.85,207.6)	−1.2 (−1.41 to −0.99)
**Eastern Sub-Saharan Africa**	4563 (3784,5347)	5.72 (4.78,6.66)	10290 (8060,12792)	5.53 (4.4,6.78)	−0.26 (−0.34 to −0.19)	3866 (3204,4552)	5.08 (4.24,5.95)	7943 (6241,9920)	4.55 (3.64,5.55)	−0.48 (−0.54 to −0.43)	120081 (98760,141884)	139.34 (115.38,164.24)	251942 (193985,318438)	123.84 (97.06,154.85)	−0.53 (−0.58 to −0.47)
**High-income Asia Pacific**	12363 (11586,13070)	6.06 (5.67,6.41)	33860 (29984,36796)	7.93 (7.15,8.54)	0.79 (0.53 to 1.05)	4522 (4179,4795)	2.28 (2.1,2.41)	11355 (9857,12346)	2.27 (2.03,2.45)	−0.23 (−0.44 to −0.02)	119985 (111637,127803)	58.35 (54.29,62.13)	218207 (196697,234504)	53.39 (48.95,57.09)	−0.51 (−0.75 to −0.28)
**High-income North America**	45582 (43959,46799)	13.68 (13.24,14.03)	71949 (67948,74894)	11.52 (10.93,11.95)	−0.61 (−0.68 to −0.54)	13725 (13135,14113)	4 (3.84,4.11)	17904 (16704,18724)	2.72 (2.55,2.85)	−1.3 (−1.43 to −1.16)	360490 (348243,371160)	110.38 (106.88,113.54)	437531 (416560,457762)	71.29 (68.07,74.46)	−1.45 (−1.59 to −1.32)
**North Africa and Middle East**	7209 (6119,8314)	4.19 (3.53,4.86)	20109 (17640,23063)	4.31 (3.79,4.93)	0.11 (0.04 to 0.17)	5608 (4722,6476)	3.44 (2.88,4)	11197 (9788,12807)	2.57 (2.26,2.94)	−0.95 (−1 to −0.9)	162945 (137353,188565)	88.12 (74.36,101.82)	314459 (272746,362189)	63.6 (55.41,73.03)	−1.09 (−1.13 to −1.05)
**Oceania**	83 (61,107)	2.66 (1.99,3.38)	237 (176,305)	2.91 (2.21,3.71)	0.4 (0.33 to 0.47)	59 (43,77)	2.11 (1.58,2.69)	165 (122,214)	2.23 (1.68,2.87)	0.31 (0.25 to 0.37)	1937 (1395,2539)	55.7 (40.86,71.84)	5322 (3875,6974)	59.18 (43.66,76.85)	0.34 (0.27 to 0.4)
**South Asia**	89166 (78180,101403)	14.24 (12.42,16.19)	259905 (224999,292158)	16.67 (14.51,18.69)	0.4 (0.29 to 0.52)	72708 (63287,83060)	12.17 (10.56,13.88)	184431 (160815,207328)	12.26 (10.73,13.76)	−0.08 (−0.15 to 0)	2309056 (2027105,2630164)	344.03 (300.12,392.47)	5542474 (4792063,6262004)	342.58 (297.07,385.82)	−0.1 (−0.17 to −0.04)
**Southeast Asia**	15356 (13322,17298)	5.87 (5.11,6.59)	45311 (38784,52437)	6.76 (5.82,7.79)	0.34 (0.29 to 0.39)	11300 (9773,12786)	4.58 (3.98,5.16)	27412 (23596,31659)	4.31 (3.73,4.96)	−0.31 (−0.34 to −0.27)	330476 (284653,375478)	117.72 (101.75,133.33)	761404 (654627,884504)	108.54 (93.48,125.86)	−0.37 (−0.4 to −0.33)
**Southern Latin America**	3861 (3541,4219)	8.28 (7.6,9.04)	4587 (4122,5059)	5.37 (4.83,5.93)	−1.28 (−1.39 to −1.17)	2667 (2458,2911)	5.76 (5.32,6.28)	2589 (2335,2847)	2.96 (2.69,3.27)	−1.95 (−2.04 to −1.85)	74951 (68698,81922)	160.06 (146.76,174.99)	64537 (58082,71436)	76.42 (68.73,84.62)	−2.26 (−2.35 to −2.16)
**Southern Sub-Saharan Africa**	1956 (1564,2394)	6.78 (5.4,8.36)	4308 (3775,4857)	6.94 (6.11,7.78)	−0.06 (−0.24 to 0.11)	1466 (1170,1810)	5.3 (4.21,6.56)	3040 (2671,3426)	5.1 (4.52,5.73)	−0.26 (−0.51 to −0.01)	45882 (36973,56193)	151.74 (121.5,186.95)	93548 (81317,106370)	144.29 (126.2,163.39)	−0.31 (−0.57 to −0.06)
**Tropical Latin America**	8491 (8095,8878)	8.84 (8.4,9.26)	22980 (21451,24403)	8.75 (8.16,9.29)	−0.09 (−0.18 to 0)	6518 (6210,6825)	7.05 (6.69,7.38)	15136 (14090,16063)	5.82 (5.41,6.17)	−0.61 (−0.7 to −0.52)	196482 (187782,205450)	194.94 (186.14,203.99)	424423 (398521,450354)	160.31 (150.47,170.03)	−0.7 (−0.81 to −0.58)
**Western Europe**	72249 (69090,75289)	13.68 (13.08,14.26)	96467 (89265,102991)	11.91 (11.11,12.69)	−0.44 (−0.51 to −0.37)	32843 (31428,34088)	6.03 (5.77,6.26)	32501 (29581,34814)	3.65 (3.36,3.9)	−1.66 (−1.77 to −1.56)	912333 (872521,949895)	177.35 (169.64,184.69)	773931 (716572,828489)	98.37 (91.68,105.14)	−1.99 (−2.07 to −1.91)
**Western SubSaharan Africa**	2193 (1753,2650)	2.4 (1.94,2.89)	5595 (4404,6835)	2.67 (2.16,3.21)	0.4 (0.36 to 0.44)	1894 (1521,2297)	2.16 (1.76,2.61)	4414 (3530,5352)	2.25 (1.85,2.69)	0.22 (0.16 to 0.28)	56426 (44779,68781)	58.15 (46.41,70.66)	133157 (103118,164195)	58.42 (46.32,71.02)	0.06 (0 to 0.11)

DALYs, disability-adjusted life-years; UI, Uncertainty Interval;EAPC, Estimated Annual Percentage Change; HNC, Head and Neck Cancer.

**Fig 1:**
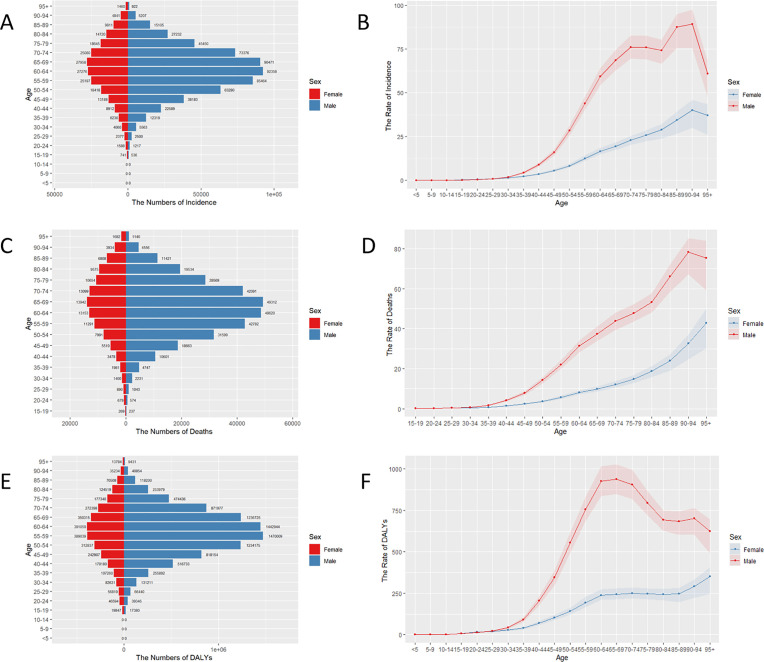
Distribution and rates of incidence, deaths, and DALYs of HNC, 2021. (A) Total new cases by age and sex. (B) ASIRs per 100,000 by age and sex. (C) Total deaths by age and sex. (D) ASDRs per 100,000 by age and sex. (E) Total DALYs by age and sex. (F) Age-standardized DALYs rates per 100,000 by age and sex. HNC, Head and neck cancer. ASIR, age-standardized incidence rate; ASDR, Age-Standardized Death Rate; DALYs, Disability-Adjusted Life Years.

The distribution of HNC incidence, deaths, and DALYs by age and sex was illustrated in Fig 1. The highest incidence rates were observed in older age groups, particularly among males. HNC predominantly affects individuals over the age of 15, with a marked increase in incidence observed between the ages of 50 and 94. The incidence rate among males remains comparatively stable in the 70–84 age group. A similar trend is evident in deaths, with a notable increase in mortality observed after the age of 84. DALYs significantly increase in the 35–69 age group, whereas a decreasing trend is observed in males over the age of 69. These findings indicated that males, particularly those in older age groups, represented a notably higher-risk population for HNC.

**Fig 2:**
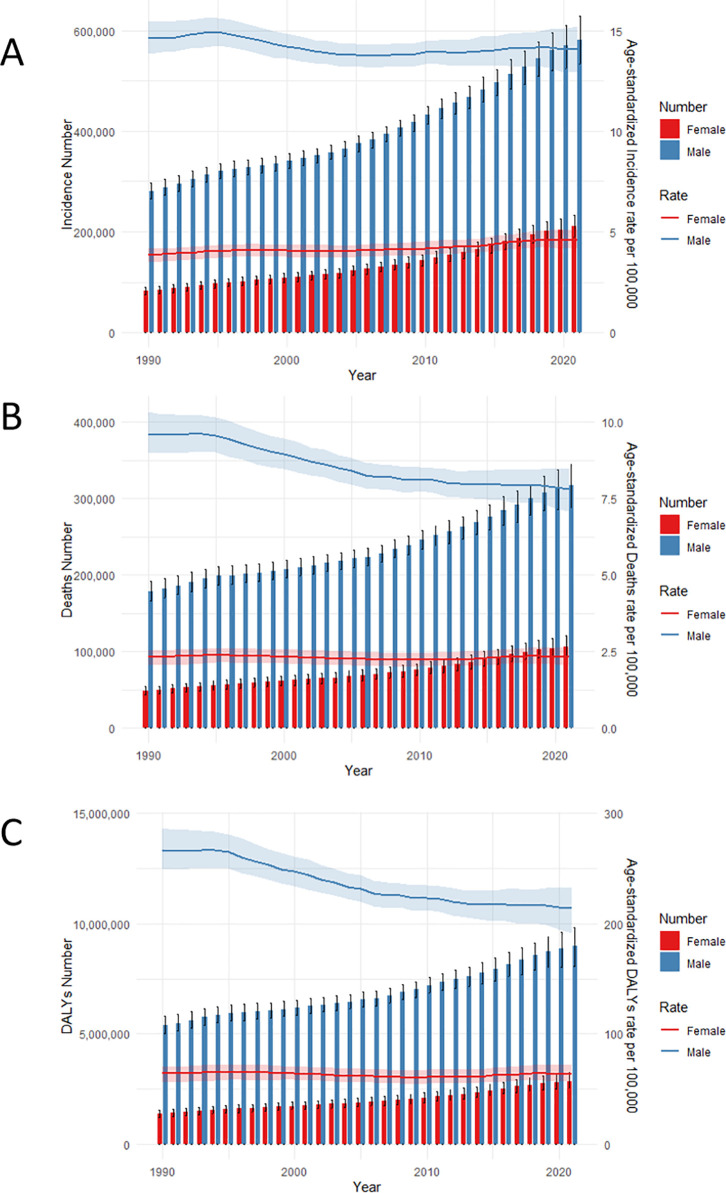
Trends in HNC metrics, differentiated by gender, from 1990 to 2021. (A) Trends in incidence and ASIRs per 100,000 for males and females. (B) Trends in deaths and ASDRs per 100,000 for males and females. (C) Trends in DALYs and Age-standardized DALYs rates per 100,000 for males and females. HNC, Head and neck cancer. ASIR, age-standardized incidence rate; ASDR, Age-Standardized Death Rate; DALYs, Disability-Adjusted Life Years.

Gender-specific trends in HNC indicators from 1990 to 2021 were presented in Fig 2. Incidence rates, mortality, and DALYs consistently increased, with males exhibiting a more pronounced rise than females. The ASIR displays a steady increase in females, while in males, it decreased from 1990 to 2006 and subsequently increased from 2006 to 2021. The ASDR continues to decline in males, while females exhibit a generally stable trend across the years. A similar declining trend in DALYs is observed in males, whereas in females, DALYs decreased from 1990 to 2009 but then increased from 2009 to 2021.

**Fig 3:**
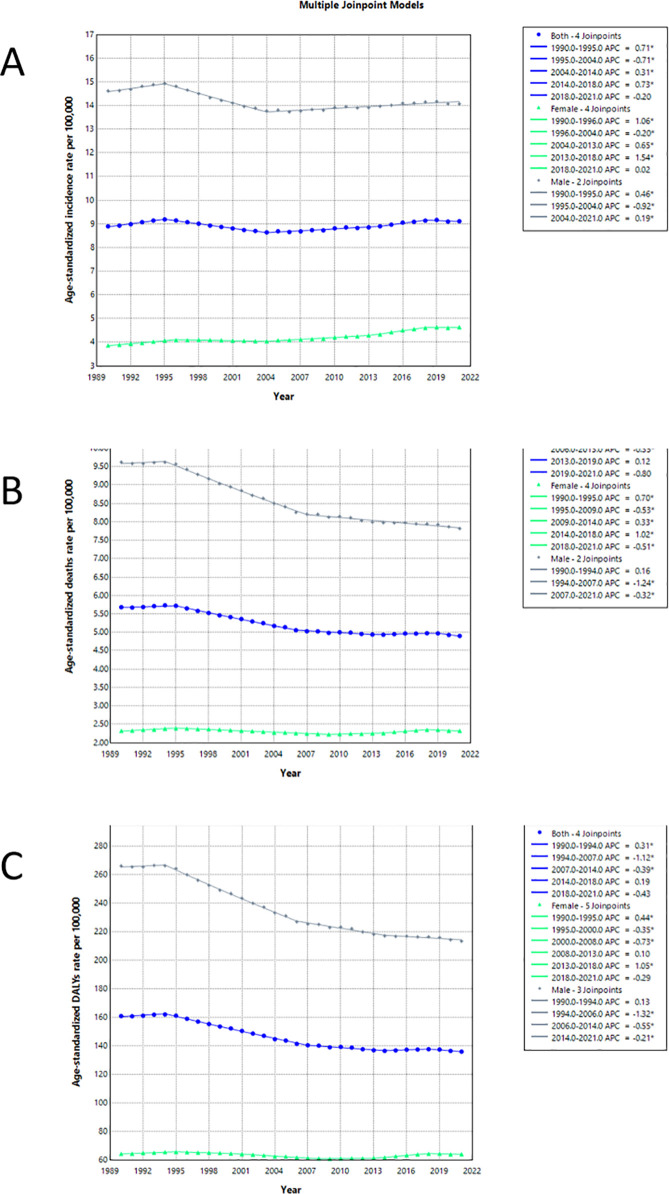
Joinpoint regression analysis of age-standardized rates for HNC from 1990 to 2021. (A) ASIRs for males (gray line) and females (green line), with the overall population trend represented by the blue line. (B) ASDRs for males, females, and the overall population. (C) Age-standardized DALYs rates for males and females, along with the total population trend. HNC, Head and neck cancer. ASIR, age-standardized incidence rate; ASDR, Age-Standardized Death Rate; DALYs, Disability-Adjusted Life Years.

Distinct global HNC trends from 1990 to 2021 were detailed in Fig 3 and S1 Table. ASIR began increasing in 2004 after a prior decline, especially among males. Although mortality rates generally decreased since 1994, they remained higher in males. Gender disparities were similarly observed in DALYs, which declined overall since approximately 1994. These observations emphasized the need for gender-specific public health interventions.

**Fig 4:**
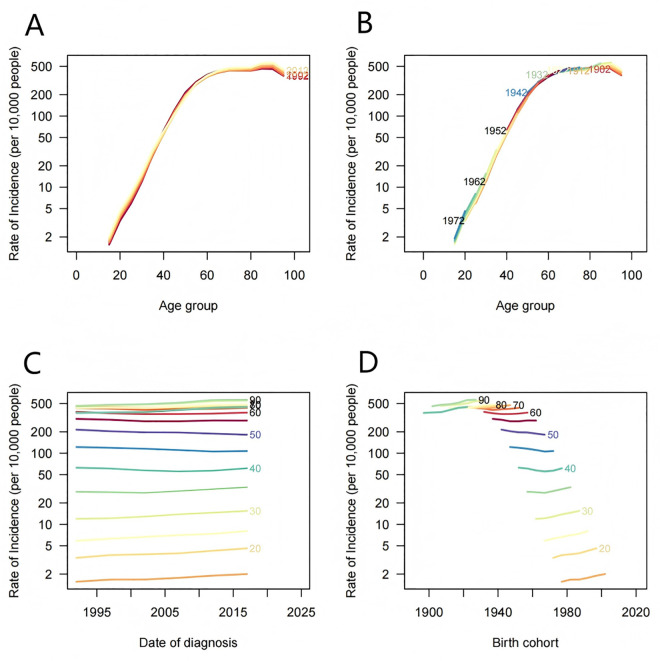
Age,period,and cohort effects on the incidence of HNC. (A) Age-specific incidence rates of HNC according to time periods;each line connects the age-specific incidence for a 5-year period. (B) Age-specific incidence rates of HNC according to birth cohort; each line connects the age-specific incidence for a 5-year cohort. (C) Period-specific incidence rates of HNC according to age groups; each line connects the birth cohort-specific incidence for a 5-year age group. (D) Birth cohort-specific incidence rates of HNC according to age groups; each line connects the birth cohort-specific incidence for a 5-year age group. HNC, Head and neck cancer.

HNC trends by age group, birth cohort, and period were illustrated in Fig 4, S1 Fig, and S2 Fig. Fluctuations in nasopharyngeal carcinoma incidence, particularly increases around 2012 for age groups 15–40 and over 70, were highlighted in Fig 4. Mortality generally declined (S1 Fig), although slight increases occurred among individuals aged 15–35 and over 80, underscoring the importance of early diagnosis. DALYs generally declined, especially among those aged 50–80 years, reflecting improved disease management but emphasizing ongoing attention (S2 Fig).

**Fig 5:**
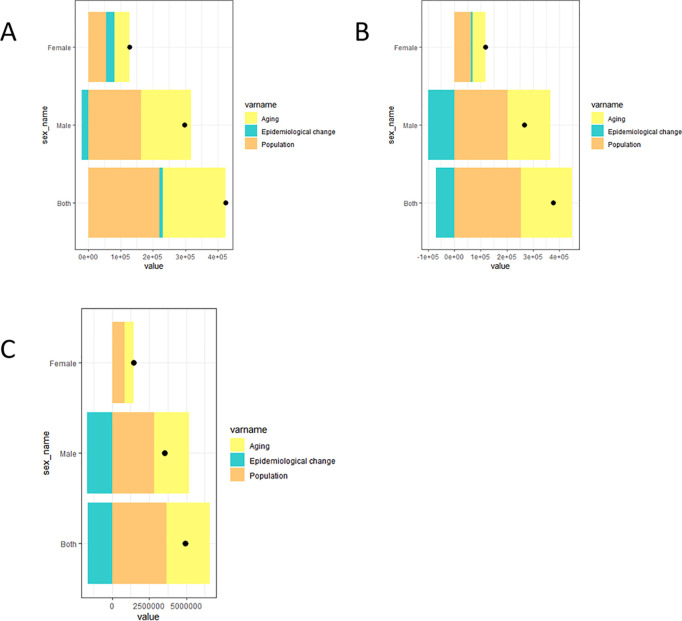
Decomposition analysis of factors contributing to HNC incidence, deaths, and DALYs from 1990 to 2021. Contributions are shown for population growth (orange), aging (yellow), and epidemiological changes (green) for both sexes, as well as for males and females. The black dots represent the cumulative effect of these three factors. (A) Decomposition of the changes in HNC incidence. (B) Decomposition of the changes in HNC deaths. (C) Decomposition of the changes in DALYs associated with HNC. HNC, Head and neck cancer. DALYs, Disability-Adjusted Life Years.

A decomposition analysis of factors influencing HNC incidence, mortality, and DALYs from 1990 to 2021 was presented in Fig 5. Epidemiological changes contributed to reducing the incidence rate among males but increased incidence among females (Fig 5A). Aging and population growth were common contributing factors to increased incidence for both genders, with a greater influence observed in males. Regarding mortality (Fig 5B), epidemiological changes similarly contributed to decreased mortality among males yet increased mortality among females. Aging and population growth consistently drove increased mortality in both genders, again with a stronger effect in males. The decomposition analysis for DALYs (Fig 5C) revealed similar patterns to those observed for mortality.

**Fig 6:**
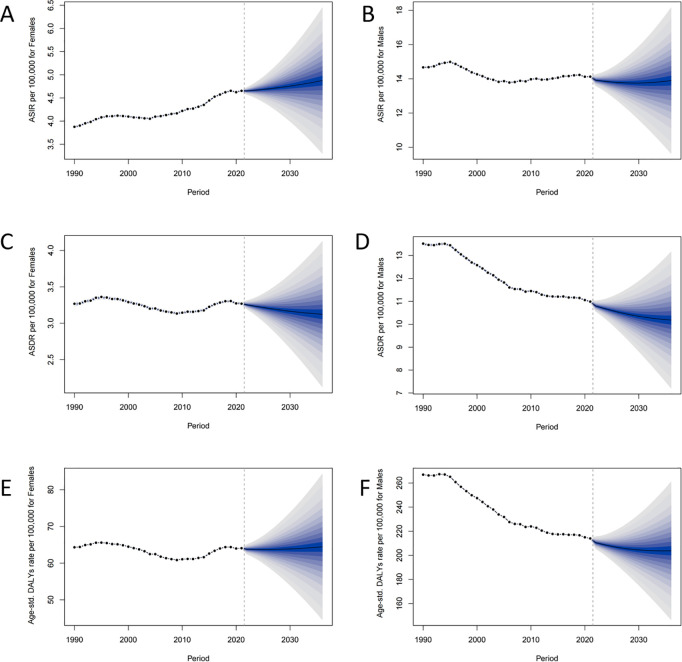
Projections of future trends in HNC metrics up to 2030. (A) Projected ASIRs per 100,000 for females. (B) Projected ASIRs per 100,000 for males. (C) Projected ASDRs per 100,000 for females. (D) Projected ASDRs per 100,000 for males. (E) Projected age-standardized DALYs rate per 100,000 for females. (F) Projected age-standardized DALYs rate per 100,000 for males. HNC, Head and neck cancer. ASIR, age-standardized incidence rate; ASDR, Age-Standardized Death Rate; DALYs, Disability-Adjusted Life Years.

Projected global trends in HNC metrics by gender up to 2030 were shown in Fig 6. ASIR was projected to significantly rise among women and remain stable among men. Both genders were anticipated to experience reductions in ASDR, with greater declines in men. DALYs were predicted to decline among men, while remaining stable among women.

**Fig 7:**
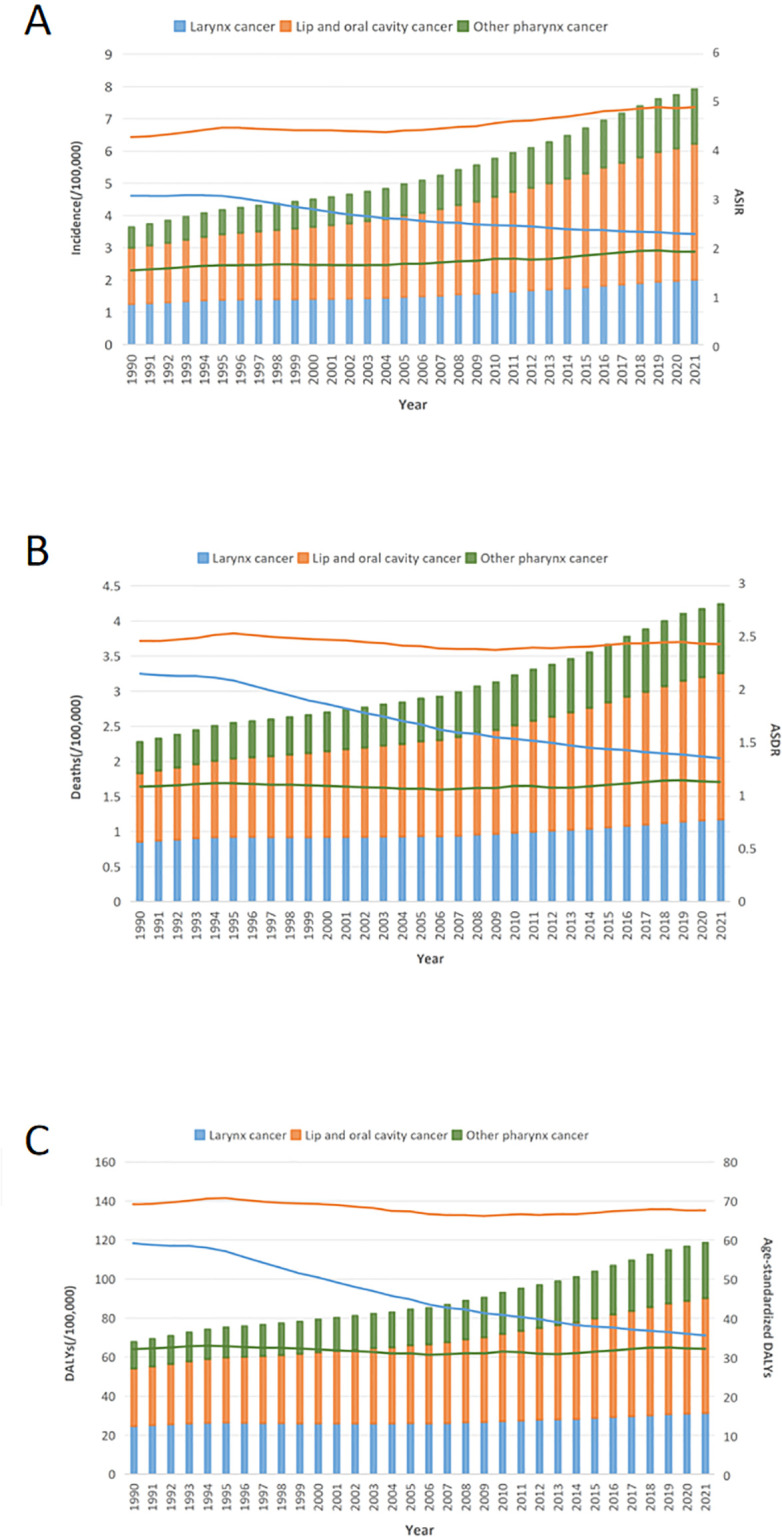
Trends in disease burden of HNC subtypes from 1990 to 2021. (A) Trends in incidence and ASIR of head and neck cancer from 1990 to 2021. (B) Trends in deaths and ASDR of head and neck cancer from 1990 to 2021. (C) Trends in DALYs and age-standardized DALYs of head and neck cancer from 1990 to 2021. HNC, Head and neck cancer. ASIR, age-standardized incidence rate; ASDR, Age-Standardized Death Rate; DALYs, Disability-Adjusted Life Years.

In 2021, Lip and Oral Cavity Cancer (LOC) exhibited the highest incidence (421,577; 95% UI: 389,879−449,782), deaths (208,379; 95% UI: 191,288−224,162), DALYs (5,874,070; 95% UI: 5,326,986−6,347,557), ASIR (4.88; 95% UI: 4.52–5.2), ASDR (2.42; 95% UI: 2.23–2.6), and age-standardized DALYs (67.71; 95% UI: 61.32–73.17) among the three subtypes of HNC (S3 Table). In contrast, Other Pharyngeal Cancer (OPC) demonstrated the lowest incidence (169,820; 95% UI: 159,847−179,704), deaths (98,435; 95% UI: 91,567−105,485), DALYs (2,843,781; 95% UI: 2,622,259−3,063,043), ASIR (1.93; 95% UI: 1.82–2.05), ASDR (1.13; 95% UI: 1.05–1.21), and age-standardized DALYs (32.38; 95% UI: 29.85–34.87) (S3 Table). The trends of incidence, deaths, DALYs, ASIR, ASDR, and age-standardized DALYs for the three subtypes of HNC from 1990 to 2021 are presented in Fig 7. LC (Larynx cancer) demonstrated a decreasing trend in ASIR, with an EAPC of −0.28 (95% CI: −0.34 to −0.22). In contrast, the other HNC subtypes exhibited increasing trends. Among these, OPC had the highest EAPC of 0.25 (95% CI: 0.15–0.34) in ASIR. OPC, as the exclusive HNC subtype, demonstrated an increasing trend in ASDR, with an EAPC of 0.04 (95% CI: −0.07 to 0.15). In contrast, its EAPC for DALYs is 0 (95% CI: −0.11 to 0.11), which is generally considered to indicate no significant change. Meanwhile, the age-standardized DALYs for LC and LOC showed a decreasing trend, with EAPC values of −0.42 (95% CI: −0.47 to −0.36) and −0.02 (95% CI: −0.14 to 0.07), respectively.

## Discussion

This study presents a thorough analysis of the trends in HNC from 1990 to 2021, offering key insights into the burden and distribution of the disease. The results indicate that, while the global incidence rate of HNC (ASIR) has remained stable, both the mortality rate (ASDR) and years lived with disability (DALYs) have exhibited a significant downward trend. These findings suggest that advances in medical technology, early diagnosis, and prevention have likely played a significant role in reducing mortality and disability-adjusted life years [2]. This phenomenon can be attributed to several potential factors: First, the widespread application of traditional multimodal treatment regimens, such as surgery, radiotherapy, and chemotherapy, has significantly improved treatment outcomes for HNC patients [8]. Second, advancements in early screening and recurrence monitoring techniques for HNC have made significant contributions to reducing the disease burden [22]. Furthermore, the ongoing development of molecular targeted therapies, immunotherapy, gene therapy, and their combined applications offers more precise and effective treatment strategies for HNC patients, suggesting a promising future for the treatment of HNC. For instance, recent studies have suggested that the expression of the UBE3C-LRP5 fusion protein may serve as a potential therapeutic target for head and neck cancer, and pyrroloquinoline quinone may emerge as a promising drug candidate for treating this malignancy [23].

This study found that the incidence, mortality, and DALYs in men are consistently higher than in women. These findings are consistent with those of Sung et al. (2021) regarding gender differences in HNC [2]. While estrogen may have protective effects against HNC [24], it is more probable that men’s higher exposure to risk factors such as smoking and alcohol consumption contributes to their increased risk of developing and dying from HNC [25]. However, the decreasing trends in mortality and DALYs suggest that treatment outcomes and survival rates have improved for men. In contrast, mortality and DALYs in women have remained relatively stable, while incidence shows a slight upward trend, suggesting that the disease burden among women is influenced by various factors. Studies suggest that changes in smoking and drinking behaviors among women may be closely associated with the increasing incidence of oropharyngeal cancer [26].Additionally, decomposition analysis indicates that population aging and growth have been the primary factors driving the increased burden of head and neck cancer over the past three decades. Predictions for 2030 further suggest that the incidence of head and neck cancer will continue to rise, particularly among women, highlighting the need for gender-specific interventions to address the disease burden in different populations. These findings underscore the ongoing and increasingly severe challenge that HNC presents to healthcare systems, particularly in high-risk populations.

The burden of HNC is rising rapidly in East Asia, with countries such as China experiencing significant increases driven by population aging and lifestyle risk factors, including tobacco and alcohol consumption [27]. Public health policies and early screening measures in East Asia may require urgent enhancement to mitigate the increasing disease burden. Similarly, Oceania has exhibited substantial annual increases in ASDR and DALYs, indicating limited progress in improving patient quality of life, consistent with recent findings by Ndon et al. [28]. Targeted public health interventions focusing on early diagnosis and timely treatment, especially among high-risk populations, are critically needed. In contrast, several countries in Central Asia and parts of Central and South America have shown declining trends in ASIR, mortality, and DALYs, likely reflecting the positive impact of strengthened public health measures and improved cancer control strategies [29].

APC analysis revealed notable trends in HNC incidence, particularly from 2002 to 2012, with the most pronounced increases occurring in the 15–35 and ≥65 age cohorts. This trend may be attributed to the increasing prevalence of HPV infections among younger populations, particularly HPV-16 [8]. Furthermore, the study indicates that HNC incidence rises with age, placing older populations at higher risk [30]. Additionally, lifestyle modifications, such as tobacco and alcohol consumption, along with greater exposure to environmental carcinogens, may collectively contribute to the observed trends. Public health policies should emphasize targeted health education, early screening, and HPV vaccination for these at-risk age groups to mitigate the incidence of HPV-positive HNSCC. Notably, mortality rates among individuals aged ≥80 have increased, potentially due to declining immune function, a higher prevalence of comorbidities, and elevated treatment-associated risks in elderly patients [31]. Therefore, comprehensive health management strategies and personalized treatment plans are essential to reducing mortality in this demographic.

Among the three subtypes of HNC analyzed, LOC present the greatest burden, with the highest incidence, mortality, and DALYs among all HNC subtypes. Compared to males, the global disease burden in females has shown a slight increase, in line with global trends [32]. Furthermore, the proportion of LOC in developed countries continues to rise [33], suggesting that early screening, diagnosis, and treatment for these cancers require further enhancement. Moreover, public awareness of risk factors such as HPV, alcohol, tobacco, betel nut consumption [34], and genetic susceptibility needs to be improved [35]. Additionally, individuals can conduct self-examinations of the oral cavity and maintain oral hygiene, which may contribute to the early detection and prevention of disease [36].The incidence of laryngeal cancer (LC) has generally declined worldwide, reflecting the success of early diagnosis and treatment strategies. In contrast, other HNC subtypes demonstrate an increasing trend, suggesting that the strategies for early detection and effective management of LC may serve as a model for other subtypes. This improvement can be attributed to robust public health interventions, tobacco control policies, and advancements in medical technology [37,38]. However, in East Asia, particularly in China, the disease burden remains on the rise [39].The incidence of OPC has been increasing globally, while mortality and DALYs have remained relatively stable. This suggests that, although early screening and diagnosis have improved, there is still significant potential for further enhancement in identifying early disease risks and exposure factors. Furthermore, advancements in medical technology, including novel treatments such as targeted therapy and immunotherapy, may have contributed to improved control of OPC-related mortality and DALYs. Despite the increasing number of new cases, patients’ quality of life and survival duration have improved. However, elderly patients may face higher risks due to declining immune function, increased comorbidities, and more severe treatment-related complications. Therefore, strengthening health management and personalized treatment plans for elderly patients is crucial for reducing mortality [8].

Despite these insights, the study has limitations. First, concerns exist regarding the accuracy and completeness of the GBD database in underdeveloped regions, as the data may not fully represent the actual disease burden. Second, the absence of data from certain regions may limit the GBD findings’ ability to comprehensively capture regional disparities in the global HNC burden. Additionally, although joinpoint regression and BAPC analysis were utilized in this study, the reliability and completeness of historical data may impact the accuracy of future projections. Besides, a major limitation of this study is the inability to directly analyze differences between HPV-positive and HPV-negative HNC cases, as the GBD 2021 database does not include HPV infection status (e.g., p16 immunohistochemistry or HPV-DNA testing data). Future GBD studies should incorporate such virological confirmation, which is crucial for evaluating vaccine policy effectiveness. Notwithstanding these data limitations, our findings, consistent with previous research, support integrating HPV vaccination into primary prevention strategies for head and neck cancer, particularly among young males and in regions with high oropharyngeal cancer (OPC) incidence.Lastly, secondary data from the GBD database may be affected by inconsistencies in definitions, reporting errors, and methodological variations, which could undermine the accuracy and comparability of the findings.

Future research should focus on assessing the burden of HNC in East Asia and among female populations, with an emphasis on environmental, lifestyle, and genetic risk factors. Advancements in early screening and personalized treatment strategies for HNC are crucial to enhancing global survival rates and quality of life. Furthermore, artificial intelligence (AI) and machine learning have shown considerable potential in diagnosing and managing HNC; thus, their integration into clinical practice should be actively advanced [40].

## Conclusions

HNC continues to pose a substantial global public health challenge, with its rising burden necessitating ongoing attention and intervention. Strategies aimed at mitigating the burden of HNC—through enhanced early diagnosis, improved access to treatment, and reduced exposure to risk factors—must consider the disparities in its global distribution. Stringent regulations on tobacco and alcohol consumption are essential for reducing the future burden of HNC. Additionally, the development, implementation, and widespread promotion of HPV vaccination constitute a fundamental preventive strategy.

## Supporting information

S1 Fig Age,period,and cohort effects on the deaths of HNC. (TIF)

S2 Fig Age,period,and cohort effects on the DALYs of HNC. (TIF)

S1 Table Joinpoint regression analysis: trends in age-standardized incidence, deaths,DALYs (per 100,000 persons) among both sexes, males, and females from 1990 to 2021 for HNC, 1990-2021. (DOCX)

S2 Table Global Burden and Trends of There Subtypes of Head and Neck Cancer from 1990 to 2021 by gender, Related to Fig 7. (DOCX)

S3 Table GBD risk hierarchy with levels. (DOCX)
