# Supplementary figures and images for: Global burden of head and neck cancer from 1990 to 2021: A comprehensive analysis and projections to 2030 based on the global burden of disease study 2021
Source: PLoS One. 2025 Sep 8;20(9):e0330805. doi: 10.1371/journal.pone.0330805 (PMC12416713; doi:10.1371/journal.pone.0330805)

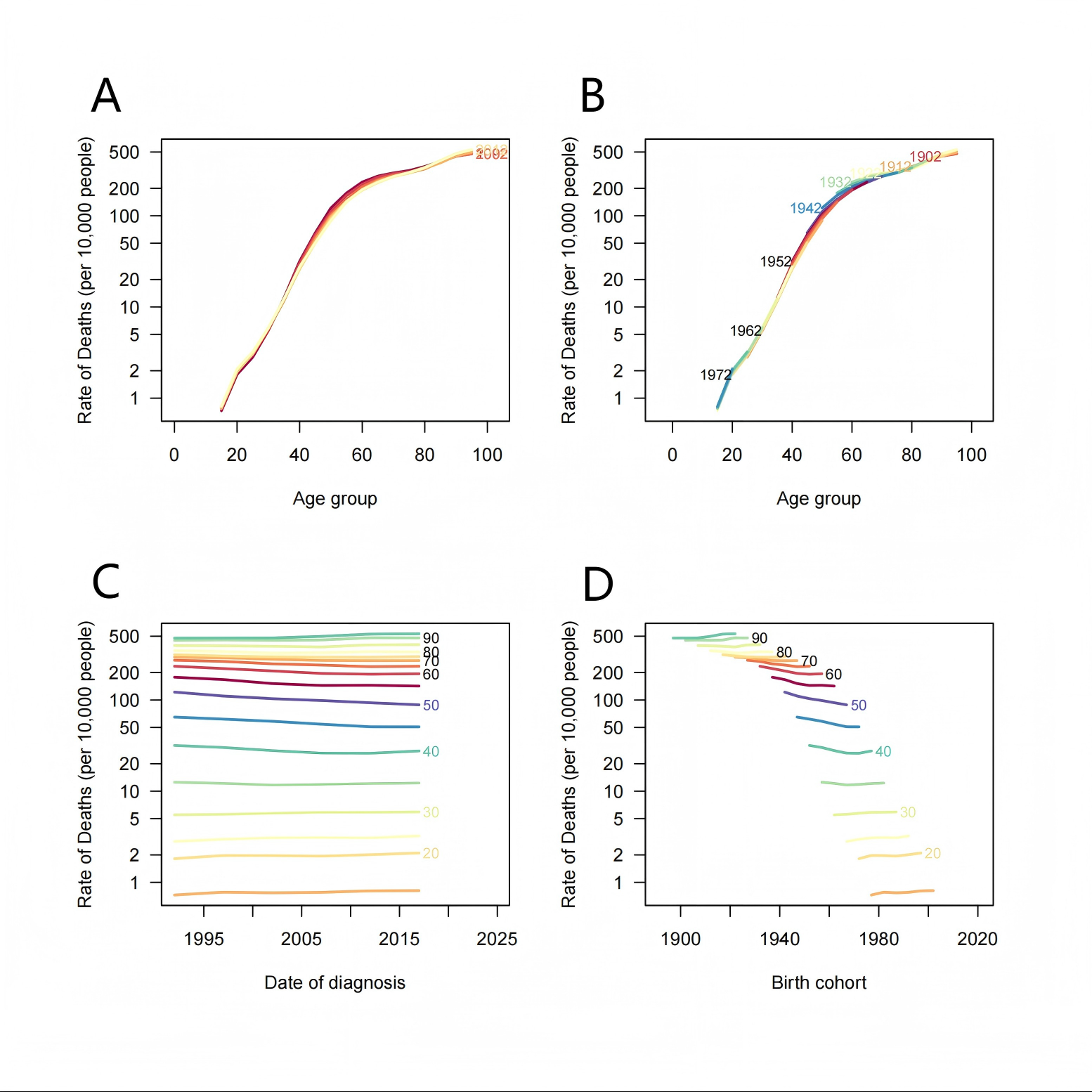

Supplement: S1 Fig — (TIF) [file pone.0330805.s001.tif]

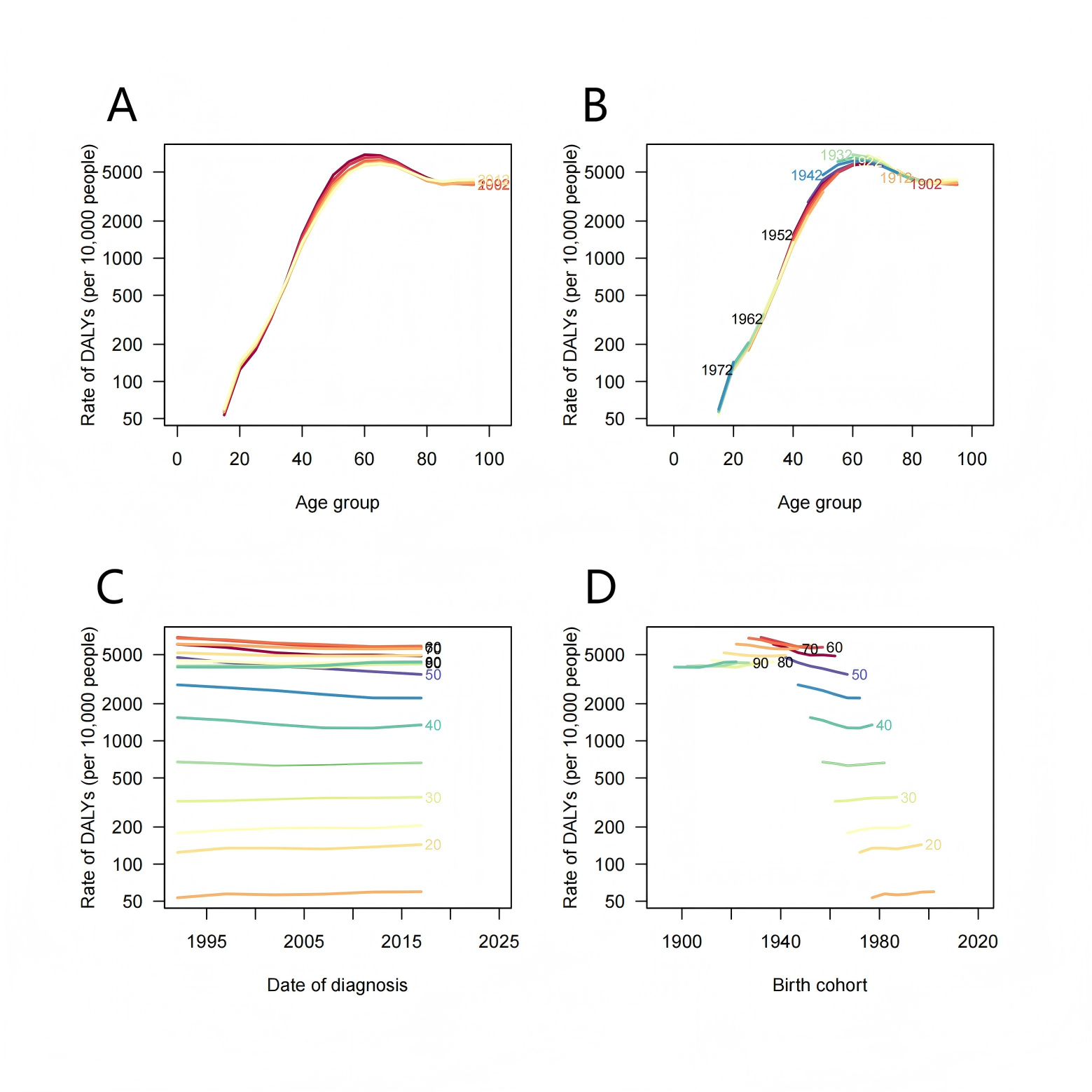

Supplement: S2 Fig — (TIF) [file pone.0330805.s002.tif]
