# Supplementary material for: Global burden of head and neck cancer from 1990 to 2021: A comprehensive analysis and projections to 2030 based on the global burden of disease study 2021
Source: PLoS One. 2025 Sep 8;20(9):e0330805. doi: 10.1371/journal.pone.0330805 (PMC12416713; doi:10.1371/journal.pone.0330805)
Supplement: S1 Table — (DOCX) [file pone.0330805.s003.docx]

| **Supplementary Table1. Joinpoint regression analysis: trends in age-standardized incidence, deaths,DALYs(per 100,000 persons) among both sexes, males, and females from 1990 to 2021 for HNC, 1990-2021.** | | | | | | | | | |
| --- | --- | --- | --- | --- | --- | --- | --- | --- | --- |
|  | Incidence |  |  | Deaths |  |  | DALYs |  |  |
| Gender | Period | APC (95% CI) | AAPC (95% CI) | Period | APC (95% CI) | AAPC (95% CI) | Period | APC (95% CI) | AAPC (95% CI) |
| Both | 1990-1995 | 0.7088(0.5287-0.8892)* | 0.0814(-0.0012-0.1641) | 1990-1995 | 0.1558(0.0086-0.3032)* | -0.4753(-0.5403--0.4102)* | 1990-1994 | 0.3123(0.0672-0.5581)* | -0.534(-0.6096--0.4584)* |
|  | 1995-2004 | -0.7057(-0.7839--0.6275)* |  | 1995-2006 | -1.104(-1.1486--1.0593)* |  | 1994-2007 | -1.1192(-1.1573--1.0811)* |  |
|  | 2004-2014 | 0.3068(0.24-0.3736)* |  | 2006-2013 | -0.353(-0.4476--0.2583)* |  | 2007-2014 | -0.3861(-0.4951--0.2769)* |  |
|  | 2014-2018 | 0.7268(0.2815-1.174)* |  | 2013-2019 | 0.1242(-0.0172-0.2659) |  | 2014-2018 | 0.1942(-0.1973-0.5873) |  |
|  | 2018-2021 | -0.1981(-0.7377-0.3443) |  | 2019-2021 | -0.7998(-1.6214-0.0287) |  | 2018-2021 | -0.4271(-0.9097-0.0578) |  |
| Female | 1990-1996 | 1.0568(0.9087-1.205)* | 0.5902(0.5113-0.6691)* | 1990-1995 | 0.7025(0.5387-0.8666)* | 0.0071(-0.0669-0.0811) | 1990-1995 | 0.4426(0.293-0.5924)* | -0.016(-0.0822-0.0501) |
|  | 1996-2004 | -0.2011(-0.3121--0.0899)* |  | 1995-2009 | -0.5289(-0.5655--0.4924)* |  | 1995-2000 | -0.347(-0.5382--0.1555)* |  |
|  | 2004-2013 | 0.6536(0.561-0.7464)* |  | 2009-2014 | 0.3255(0.0977-0.5539)* |  | 2000-2008 | -0.7251(-0.8033--0.6469)* |  |
|  | 2013-2018 | 1.5387(1.2492-1.8291)* |  | 2014-2018 | 1.0158(0.6375-1.3956)* |  | 2008-2013 | 0.1016(-0.095-0.2987) |  |
|  | 2018-2021 | 0.0153(-0.51-0.5433) |  | 2018-2021 | -0.5069(-0.9213--0.0907)* |  | 2013-2018 | 1.0481(0.8488-1.2478)* |  |
|  |  |  |  |  |  |  | 2018-2021 | -0.2882(-0.6458-0.0707) |  |
| Male | 1990-1995 | 0.4569(0.2331-0.6811)* | -0.0935(-0.1403--0.0466)* | 1990-1994 | 0.1595(-0.1818-0.502) | -0.6462(-0.6972--0.5953)* | 1990-1994 | 0.129(-0.176-0.435) | -0.687(-0.7444--0.6296)* |
|  | 1995-2004 | -0.9232(-1.0158--0.8304)* |  | 1994-2007 | -1.2424(-1.2952--1.1895)* |  | 1994-2006 | -1.3223(-1.3744--1.2701)* |  |
|  | 2004-2021 | 0.1866(0.1498-0.2233)* |  | 2007-2021 | -0.3197(-0.3676--0.2718)* |  | 2006-2014 | -0.5549(-0.6549--0.4547)* |  |
|  |  |  |  |  |  |  | 2014-2021 | -0.2086(-0.3563--0.0606)* |  |
| HNC, Head and Neck Cancer; AAPC, average annual percent change presented for full period; APC, annual percent change; CI, confidence interval. *, P<0.05 | | | | | | | | | |
|  |  |  |  |  |  |  |  |  |  |
