# Supplementary material for: Global burden of head and neck cancer from 1990 to 2021: A comprehensive analysis and projections to 2030 based on the global burden of disease study 2021
Source: PLoS One. 2025 Sep 8;20(9):e0330805. doi: 10.1371/journal.pone.0330805 (PMC12416713; doi:10.1371/journal.pone.0330805)
Supplement: S2 Table — (DOCX) [file pone.0330805.s004.docx]

| **Supplementary Table2 . GBD risk hierarchy with levels** | |
| --- | --- |
| **level** | **Risk** |
| 0 | All risk factors |
| 1 | Environmental/occupational risks |
| 2 | Unsafe water, sanitation, and handwashing |
| 3 | Unsafe water source |
| 3 | Unsafe sanitation |
| 3 | No access to handwashing facility |
| 2 | Air pollution |
| 3 | Particulate matter pollution |
| 4 | Ambient particulate matter pollution |
| 4 | Household air pollution from solid fuels |
| 3 | Ambient ozone pollution |
| 3 | Nitrogen dioxide pollution |
| 2 | Non-optimal temperature |
| 3 | High temperature |
| 3 | Low temperature |
| 2 | Other environmental risks |
| 3 | Residential radon |
| 3 | Lead exposure |
| 2 | Occupational risks |
| 3 | Occupational carcinogens |
| 4 | Occupational exposure to asbestos |
| 4 | Occupational exposure to arsenic |
| 4 | Occupational exposure to benzene |
| 4 | Occupational exposure to beryllium |
| 4 | Occupational exposure to cadmium |
| 4 | Occupational exposure to chromium |
| 4 | Occupational exposure to diesel engine exhaust |
| 4 | Occupational exposure to formaldehyde |
| 4 | Occupational exposure to nickel |
| 4 | Occupational exposure to polycyclic aromatic hydrocarbons |
| 4 | Occupational exposure to silica |
| 4 | Occupational exposure to sulfuric acid |
| 4 | Occupational exposure to trichloroethylene |
| 3 | Occupational asthmagens |
| 3 | Occupational particulate matter, gases, and fumes |
| 3 | Occupational noise |
| 3 | Occupational injuries |
| 3 | Occupational ergonomic factors |
| 1 | Behavioral risks |
| 2 | Child and maternal malnutrition |
| 3 | Suboptimal breastfeeding |
| 4 | Non-exclusive breastfeeding |
| 4 | Discontinued breastfeeding |
| 3 | Child growth failure |
| 4 | Child underweight |
| 4 | Child wasting |
| 4 | Child stunting |
| 3 | Low birth weight and short gestation |
| 4 | Short gestation |
| 4 | Low birth weight |
| 3 | Iron deficiency |
| 3 | Vitamin A deficiency |
| 3 | Zinc deficiency |
| 2 | Tobacco |
| 3 | Smoking |
| 3 | Chewing tobacco |
| 3 | Secondhand smoke |
| 2 | High alcohol use |
| 2 | Drug use |
| 2 | Dietary risks |
| 3 | Diet low in fruits |
| 3 | Diet low in vegetables |
| 3 | Diet low in legumes |
| 3 | Diet low in whole grains |
| 3 | Diet low in nuts and seeds |
| 3 | Diet low in milk |
| 3 | Diet high in red meat |
| 3 | Diet high in processed meat |
| 3 | Diet high in sugar-sweetened beverages |
| 3 | Diet low in fiber |
| 3 | Diet low in calcium |
| 3 | Diet low in seafood omega-3 fatty acids |
| 3 | Diet low in omega-6 polyunsaturated fatty acids |
| 3 | Diet high in trans fatty acids |
| 3 | Diet high in sodium |
| 2 | Intimate partner violence |
| 2 | Childhood sexual abuse and bullying |
| 3 | Childhood sexual abuse |
| 3 | Bullying victimization |
| 2 | Unsafe sex |
| 2 | Low physical activity |
| 1 | Metabolic risks |
| 2 | High fasting plasma glucose |
| 2 | High LDL cholesterol |
| 2 | High systolic blood pressure |
| 2 | High body-mass index |
| 2 | Low bone mineral density |
| 2 | Kidney dysfunction |
