# Supplementary material for: Global burden of head and neck cancer from 1990 to 2021: A comprehensive analysis and projections to 2030 based on the global burden of disease study 2021
Source: PLoS One. 2025 Sep 8;20(9):e0330805. doi: 10.1371/journal.pone.0330805 (PMC12416713; doi:10.1371/journal.pone.0330805)
Supplement: S3 Table — (DOCX) [file pone.0330805.s005.docx]

| **Supplementary Table3. Global Burden and Trends of There Subtypes of Head and Neck Cancer from 1990 to 2021 by gender, Related to Figure 7** | | | | | | | | | | | | | | | | |
| --- | --- | --- | --- | --- | --- | --- | --- | --- | --- | --- | --- | --- | --- | --- | --- | --- |
| **HNC subtypes** | **Gender** | 1990 | | 2021 | |  | 1990 |  | 2021 |  |  | 1990 |  | 2021 |  |  |
|  |  | Incidence cases | ASIR | Incidence cases | ASIR | EAPC | Death cases | ASDR | Death cases | ASDR | EAPC | DALYs cases | Age_standardised DALYs | DALYs cases | Age_standardised DALYs | EAPC |
|  |  | (95%UI) | per 100,000(95%UI) | (95%UI) | per 100,000(95%UI) | (95%UI) | (95%UI) | per 100,000(95%UI) | (95%UI) | per 100,000(95%UI) | (95%UI) | (95%UI) | per 100,000(95%UI) | (95%UI) | per 100,000(95%UI) | (95%UI) |
| Larynx cancer | Both | 125175 (118981,131639) | 3.07 (2.92,3.23) | 200883 (186941,216098) | 2.29 (2.13,2.47) | -0.28 (-0.34,-0.22) | 85790 (80409,91208) | 2.15 (2.01,2.28) | 117252 (109355,125952) | 1.35 (1.26,1.45) | -0.4 (-0.45,-0.34) | 2475842 (2313814,2636993) | 59.3 (55.47,63.1) | 3143309 (2922792,3383514) | 35.8 (33.29,38.54) | -0.42 (-0.47,-0.36) |
|  | Female | 15297 (12506,16734) | 0.71 (0.58,0.78) | 29094 (24975,33680) | 0.63 (0.54,0.73) | -0.11 (-0.21,0.09) | 10528 (8183,11773) | 0.49 (0.39,0.55) | 16859 (14209,19876) | 0.37 (0.31,0.43) | -0.26 (-0.35,-0.08) | 294696 (220024,332508) | 13.49 (10.13,15.19) | 449130 (377885,540000) | 9.93 (8.33,11.93) | -0.26 (-0.37,-0.06) |
|  | Male | 109879 (104446,116063) | 5.8 (5.5,6.12) | 171789 (159470,186042) | 4.16 (3.86,4.5) | -0.25 (-0.31,-0.19) | 75262 (70749,80447) | 4.12 (3.87,4.4) | 100393 (93351,108830) | 2.49 (2.31,2.69) | -0.37 (-0.43,-0.32) | 2181146 (2048786,2333042) | 110.04 (103.4,117.38) | 2694179 (2491890,2926037) | 64.13 (59.35,69.57) | -0.4 (-0.45,-0.34) |
| Lip and oral cavity cancer | Both | 174077 (167404,181622) | 4.27 (4.1,4.45) | 421577 (389879,449782) | 4.88 (4.52,5.2) | 0.14 (0.04,0.22) | 97402 (92506,102361) | 2.45 (2.33,2.58) | 208379 (191288,224162) | 2.42 (2.23,2.6) | -0.01 (-0.12,0.07) | 2936205 (2793741,3092058) | 69.27 (65.92,72.96) | 5874070 (5326986,6347557) | 67.71 (61.32,73.17) | -0.02 (-0.14,0.07) |
|  | Female | 55690 (52276,59042) | 2.59 (2.43,2.74) | 148660 (135704,160404) | 3.28 (3,3.54) | 0.26 (0.17,0.38) | 30412 (28096,32662) | 1.45 (1.34,1.55) | 71489 (64699,78443) | 1.56 (1.42,1.71) | 0.08 (-0.02,0.2) | 870962 (803992,939990) | 39.59 (36.55,42.72) | 1904258 (1730274,2088527) | 42.58 (38.63,46.78) | 0.08 (-0.04,0.2) |
|  | Male | 118387 (112777,124445) | 6.17 (5.88,6.49) | 272917 (245321,296016) | 6.65 (5.99,7.21) | 0.08 (-0.06,0.18) | 66990 (62782,71608) | 3.63 (3.4,3.89) | 136890 (120656,149372) | 3.39 (3,3.69) | -0.07 (-0.21,0.03) | 2065244 (1932213,2212857) | 101.41 (94.96,108.49) | 3969812 (3446429,4348773) | 94.55 (82.23,103.44) | -0.07 (-0.22,0.04) |
| Other pharynx cancer | Both | 64529 (60829,68920) | 1.55 (1.46,1.66) | 169820 (159847,179704) | 1.93 (1.82,2.05) | 0.25 (0.15,0.34) | 44344 (40994,48190) | 1.08 (1,1.18) | 98435 (91567,105485) | 1.13 (1.05,1.21) | 0.04 (-0.07,0.15) | 1372478 (1270671,1494393) | 32.3 (29.92,35.17) | 2843781 (2622259,3063043) | 32.38 (29.85,34.87) | 0 (-0.11,0.11) |
|  | Female | 12012 (10617,13743) | 0.56 (0.49,0.63) | 32753 (28867,39597) | 0.72 (0.63,0.87) | 0.29 (0.13,0.48) | 8173 (6970,9702) | 0.38 (0.33,0.45) | 17998 (15452,22431) | 0.39 (0.34,0.49) | 0.03 (-0.12,0.21) | 243179 (203246,292981) | 11.06 (9.28,13.33) | 509884 (440655,635095) | 11.38 (9.8,14.19) | 0.03 (-0.15,0.22) |
|  | Male | 52517 (49128,56661) | 2.64 (2.47,2.85) | 137066 (128148,146458) | 3.26 (3.05,3.48) | 0.23 (0.12,0.34) | 36171 (33086,39869) | 1.87 (1.71,2.05) | 80437 (73959,87131) | 1.94 (1.79,2.1) | 0.04 (-0.09,0.16) | 1129299 (1033227,1247782) | 54.85 (50.2,60.59) | 2333897 (2128450,2539518) | 54.77 (50,59.57) | 0 (-0.13,0.13) |
| DALYs, disability-adjusted life-years；UI，Uncertainty Interval;EAPC，Estimated Annual Percentage Change；HNC, Head and Neck Cancer.ASIR, age-standardized incidence rate; ASDR, Age-Standardized Death Rate. | | | | | | | | | | | | | | | | |
